# Targeting immunosenescence and inflammaging: advancing longevity research

**DOI:** 10.1038/s12276-025-01527-9

**Published:** 2025-09-01

**Authors:** Thi Quynh Trang Nguyen, Kyung A Cho

**Affiliations:** 1 Department of Biochemistry, Chonnam National University Medical School, Hwasun-gun, Republic of Korea; 2 Center for Creative Biomedical Scientists, Chonnam National University Medical School, Hwasun-gun, Republic of Korea; 3 MediSpan Inc., Bundang-gu, Republic of Korea

**Keywords:** Biological therapy, Translational immunology, Immunological techniques, Drug development

## Abstract

Here we examine the crucial role of the immune system in aging, with a particular focus on two interconnected processes: immunosenescence and inflammaging, which contribute to age-related decline. Our goal is to provide a thorough overview of the various factors that lead to immune aging while introducing therapeutic approaches that can partially restore immune function. Additionally, we discuss recent strategies that go beyond localized immune improvement to actively modulate immune balance, influencing systemic aging and extending healthspan. Through this exploration, we propose that regulating the immune system is essential for managing immune aging and may serve as a key mechanism for controlling the overall aging process and promoting healthy longevity.

## Modulating immunity to combat aging and extend healthspan

Aging affects our immune system, leading to a decline in its function and increased inflammation, known as immunosenescence and inflammaging. Researchers have studied ways to improve immune health and extend lifespan. It reviews various strategies to combat immune aging, such as rejuvenating the thymus, enhancing stem cell function and using therapies to clear senescent cells. These approaches aim to restore immune balance and reduce chronic inflammation. It highlights the importance of targeting specific pathways, such as IL-11 and TLR5, to improve immune resilience and promote healthy aging. The findings suggest that by addressing both immune decline and chronic inflammation, we can potentially extend lifespan and improve health in older adults. Future research will focus on refining these strategies for clinical use, offering hope for healthier aging.

This summary was initially drafted using artificial intelligence, then revised and fact-checked by the author.

## Introduction

Aging is a multifaceted biological process that profoundly impacts immune system function. Innate and adaptive immunity declines mark this process, collectively termed immune aging, which includes two interconnected phenomena: immunosenescence and inflammaging. These processes represent opposing yet complementary facets of immune aging, where a decline in beneficial immune functions coexists with chronic, maladaptive inflammation^1,2^. Immunosenescence is the immune system’s progressive functional decline, impairing innate and adaptive immunity. Hallmarks include thymic involution, reduced output of naive T cells, diminished immune surveillance and a weakened capacity for proinflammatory responses critical for pathogen detection and clearance^3^. Adaptive immunity suffers from an accumulation of memory T and B cells at the expense of naive populations, leading to a contraction of the overall T cell receptor repertoire diversity and a heightened risk of autoimmunity. Similarly, innate immune functions, such as natural killer (NK) cell activity and macrophage responsiveness, are compromised, further weakening the immune response^4^. Inflammaging, on the other hand, describes the persistent, low-grade systemic inflammation characteristic of aging. Unlike the acute, protective inflammation necessary for tissue repair and immune activation, inflammaging arises from the chronic activation of immune cells by senescence-associated secretory phenotype (SASP) factors, damage-associated molecular patterns (DAMPs), and dysregulated gut microbiota. This chronic inflammation exacerbates tissue damage, impairs cellular function and accelerates the progression of age-related diseases such as cardiovascular disorders and neurodegeneration^5^. While protective inflammation and immune activation decline, inappropriate chronic inflammation persists, creating an immune environment prone to dysfunction and disease. This paradox underpins the dual challenges of aging immunity: a diminished capacity to respond effectively to pathogens and a maladaptive inflammatory state. The coronavirus disease 2019 (COVID-19) pandemic has further underscored the consequences of immune aging. With their diminished ability to mount effective immune responses, older populations experienced disproportionately severe outcomes. These events have catalyzed a renewed focus on understanding and addressing immunosenescence and inflammaging^6^. Thus, this Review Article highlights advancements in understanding immune aging, approaches to mitigate its effects and novel technologies that directly modulate immunity to extend lifespan. We aim to demonstrate how immunosenescence and inflammaging have evolved from being markers of decline to pivotal targets for therapeutic intervention. This perspective positions immune modulation as a cornerstone of future aging and longevity therapies, potentially transforming how we approach age-related diseases and healthspan extension^7–9^.

## Mechanisms and conceptual framework of immunosenescence: interplay between immunosenescence and inflammaging

**Fig. 1:**
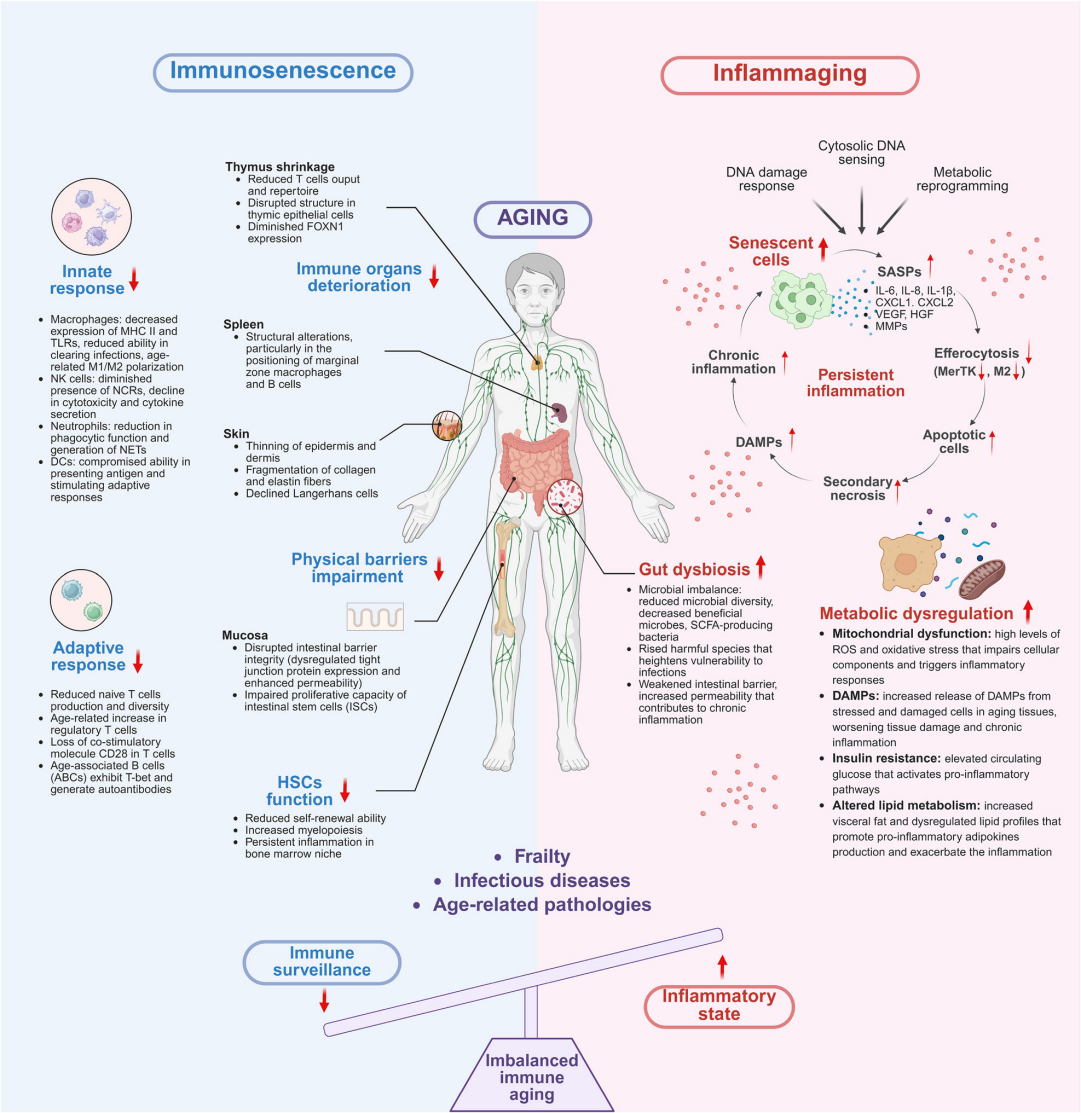
Immunosenescence and inflammaging: dual pillars of aging-related immune dysfunction. This schematic illustrates the two major immune-aging axes—immunosenescence and inflammaging—and their systemic impact on organismal aging. Immunosenescence is marked by deterioration of immune organs (for example, thymus, spleen and skin), impaired physical and mucosal barriers, reduced HSC function and weakened innate and adaptive immune responses. In parallel, inflammaging arises from persistent inflammation driven by senescent cell accumulation, SASP secretion, metabolic dysregulation, mitochondrial dysfunction and gut dysbiosis. Together, these processes promote immune imbalance, leading to increased vulnerability to infections, frailty and age-related diseases. The figure was created with BioRender.com.

Aging induces complex changes in the immune system, driven by weakened physical barriers, the decline of immune organs and heightened inflammatory responses. These factors compromise immune surveillance and foster the development of frailty and age-related diseases (Fig. 1). This section provides an overview of these interconnected mechanisms to establish the foundation for immune-targeted interventions.

### Immunosenescence: decline in immune systems

The skin and intestinal barriers are critical first-line defenses in the innate immune system. However, aging causes structural and functional deterioration in these barriers, rendering them less effective in preventing microbial invasion and maintaining immune homeostasis.

#### Physical barrier weakening

Aging causes structural changes in physical barriers such as the skin and intestinal epithelium, impairing their ability to protect against pathogens and maintain immune homeostasis. In the skin, epidermal and dermal thinning and fragmented collagen and elastin fibers weaken the barrier. At the same time, a reduction in epidermis-resident dendritic cells (DCs) (Langerhans cells) and an increase in Foxp3^+^ regulatory T cells (T_reg_) compromise antigen-specific immunity and contribute to infection susceptibility in the elderly^10^. In the intestinal barriers, altered tight-junction protein expression and increased permeability disrupt immune surveillance and promote inflammation. Higher claudin-2 levels in aging humans and baboons facilitate macromolecule permeability. At the same time, reduced colonic mucus layer thickness and microbiota changes in aged mice lead to decreased T cell signaling and impaired mucosal immunity^11,12^. Additionally, intestinal stem cells show reduced proliferation and self-renewal, limiting mucosal repair and weakening epithelial defenses against pathogens^13^. These barrier defects exacerbate systemic inflammation and compromise immune function, further accelerating immunosenescence. Similar age-related changes in barrier integrity are observed in the lung, kidney and eyes, highlighting their role in systemic immune decline^14^.

#### Immune organ decline

Aging profoundly affects primary and secondary immune organs, diminishing their ability to support effective immune responses. The thymus is particularly affected, undergoing involution that leads to decreased production and diversity of T lymphocytes, impairing immune responsiveness. Among various thymic cell types, thymic epithelial cells (TECs), including cortical and medullary TECs, are essential for differentiating T cells. Histological examination of the aging thymus reveals a disorganized architecture with decreased TECs and obscure boundaries between the thymus cortex and medulla, occupied by adipocytes and fibroblasts^15^. FOXN1, a master transcriptional regulator of TEC biology, declines with age and contributes to thymic involution^16^. Recently, a study reported that atypical age-associated TECs form high-density clusters lacking thymocytes, further contributing to the loss of functional thymic tissue. These age-associated TECs are driven by the loss of FOXN1 expression^17^. The spleen, a secondary lymphoid organ, also undergoes age-related structural changes. Disrupted localization and organization of marginal zone macrophages and marginal zone B cells impair the surveillance of blood-derived antigens^18^. Hematopoietic stem cells (HSCs) exhibit important changes in function with aging, including impaired self-renewal capacity and a shift toward increased myelopoiesis^19,20^. Myeloid-biased phenotypes of aged HSC are characterized by the upregulation of myeloid-biased marker CD150 and the downregulation of lymphoid-biased marker CD86 (ref. ^21^). Chronic inflammation within the bone marrow niche, driven by elevated levels of IL-1β, TNF, IL-6 and TGF-β1, further exacerbates these dysfunctions^21^.

#### Innate immunity

With age, the innate immune system undergoes several critical changes. The expression of key innate immune receptors, such as Toll-like receptors (TLRs) and NOD-like receptors (NLRs), is noticeably reduced, compromising the detection and clearance of pathogens. This impaired function diminishes the body’s ability to mount effective immune responses to infections, contributing to increased susceptibility to microbial invasion^22,23^. Simultaneously, the decline in receptor expression and signaling leads to a dysregulated cytokine response characterized by elevated inflammatory mediators, which can exacerbate chronic inflammation and tissue damage^24^. Macrophages, crucial for pathogen recognition and phagocytosis, exhibit diminished capacity to eliminate pathogens. This decline is attributed to reduced surface expression of essential markers such as MHC class II and TLRs^22–24^. Age-related M1/M2 polarization shifts are tissue-dependent, with M1-like macrophages more prevalent in the liver and adipose tissue and M2-like macrophages in the bone marrow, lymphoid tissues, spleen, muscle and lungs^3,25^. NK cells, responsible for detecting and destroying virally infected and malignant cells, display reduced cytotoxicity and cytokine production with age, compromising the body’s ability to control infections and prevent tumor formation^26^. While circulating NK cells increase in number with age, their functional efficiency declines due to altered receptor expression, including decreased natural cytotoxicity receptors (NCRs), such as NKp30 and NKp46 (refs. ^27,28^). Neutrophils from older adults show diminished phagocytic activity and reduced generation of neutrophil extracellular traps for capturing pathogens^29,30^. DCs, despite no pronounced evidence of age-related changes in numbers, exhibit impaired antigen-presenting capacity. Aged conventional DCs (cDCs) have reduced ability to activate CD8^+^ T cell-driven cytotoxic responses, and plasmacytoid DCs produce less IFN-α in response to TLR7 and TLR9 ligands, increasing susceptibility to viral infections^31,32^.

#### Adaptive immunity

Adaptive immunity, crucial for long-term immune responses and memory, undergoes profound aging changes impairing efficacy. T cells exhibit well-documented age-related changes, including reduced naive T cell production and repertoire diversity due to thymic involution^33–36^. T_reg_ increase with age, potentially suppressing effective immune responses^33^. CD8^+^ T cells experience marked depletion of naive populations and accumulate highly differentiated CD28^−^ effector memory T cells, leading to impaired responses to new antigens^34,35^. Moreover, the expansion of CD28^−^ T cells with age contributes to immune dysfunction through impaired proliferative responses, altered cytokine secretion and the expression of senescence or exhaustion markers such as CD57 and KLRG1. These changes limit the adaptive immune system’s ability to effectively respond to novel infections and vaccinations^36–38^. B cell production and functionality also decline with age. Aged B cells exhibit senescence markers and produce less effective antibodies, impairing humoral immune responses and vaccine efficacy^39,40^. Accumulation of age-associated B cells, which express T-bet for autoantibody production, may contribute to autoimmune diseases^39^. Dysregulated B cell activity, combined with impaired antigen presentation by DCs, reduces the efficacy of both natural and vaccine-induced immunity^41,42^.

### Inflammaging: increased inflammation with aging

Aging is associated with chronic, low-grade inflammation, commonly called ‘inflammaging’. This persistent inflammatory state is driven by the accumulation of senescent cells and their associated secretory phenotype (SASP), metabolic dysregulation and alterations in the gut microbiome.

#### SASP

Cellular senescence, initially identified as a protective mechanism to prevent the proliferation of damaged or stressed cells, has emerged as a key contributor to the chronic inflammation observed in aging, commonly referred to as ‘inflammaging’^43^. Senescent cells secrete a complex matrix components, promoting tissue structural alterations that facilitate tumor mixture of proinflammatory and bioactive molecules collectively known as the SASP^44^. This persistent secretion alters the tissue microenvironment, exacerbates immune dysfunction and contributes to the progression of age-related diseases^45^. The secreted SASP components collectively disrupt the tissue microenvironment and amplify inflammatory responses: proinflammatory cytokines and chemokines such as IL-6, IL-8, IL-1β, CXCL1 and CXCL2 recruit immune cells and amplify inflammation. However, prolonged exposure can lead to tissue damage and immune system exhaustion^46^. Growth factors such as VEGF and HGF stimulate cell proliferation and angiogenesis but may also contribute to tissue fibrosis or tumorigenesis^47^. Proteases such as matrix metalloproteinases degrade extracellular progression. These components, secreted in an unregulated manner, exacerbate chronic inflammation, impair immune surveillance and promote tissue dysfunction, thereby driving the development of age-related diseases, including cancer, osteoarthritis and neurodegeneration^44^.

The SASP is not merely a bystander but a dynamic regulator of tissue homeostasis, with its composition shaped by cell type, senescence trigger and microenvironmental context^48,49^. SASP is primarily orchestrated by DNA damage response pathways and cytosolic DNA sensing. Persistent DNA damage activates transcription factors NF-κB and C/EBPβ to upregulate proinflammatory cytokines (IL-6, IL-8 and IL-1α), which can reinforce their own production via autocrine loop, thereby intensifying SASP output and propagating senescence to neighboring cells^48,50^. In parallel, cytosolic DNA fragments including mitochondrial DNA and cytoplasmic chromatic trigger the cGAS-STING pathway, driving interferon-stimulated genes such as CXCL10 and CCL5, which recruit immune cells and sustain inflammation^48,51^. Metabolic reprogramming in senescent cells, including NAD⁺ depletion and mitochondrial oxidative stress, further potentiate SASP by suppressing AMPK and enhancing mTOR/NF-κB signaling^48,52^. Together, these interconnected pathways form an integrated network that perpetuates SASP-driven chronic inflammation.

Furthermore, SASP factors such as IL-6, TGF-β and TNF can disrupt macrophage polarization and efferocytosis-the critical process by which macrophages eliminate apoptotic cells^53,54^. Specifically, SASP skews macrophages toward a proinflammatory M1 phenotype while suppressing reparative M2 polarization, impairing their ability to resolve inflammation^53^. Age-related impairment of efferocytosis, driven by diminished MerTK signaling^55,56^, reduced autophagy^57^ and mitochondrial dysfunction^58^, allow apoptotic debris to accumulate and undergo secondary necrosis, then release DAMPs that amplify the inflammatory cascades. This unresolved inflammation further exacerbates mitochondrial stress and cellular senescence, creating a vicious cycle, which promotes chronic inflammation, compromises tissue homeostasis and fuels age-related pathologies such as fibrosis and atherosclerosis^55,56^. Therefore, targeting efferocytosis or neutralizing SASP components may disrupt this cycle, offering strategies to mitigate inflammaging and its systemic consequences.

#### Metabolic dysregulation

Metabolic dysregulation is a major driver of inflammaging, contributing to the chronic low-grade inflammation associated with aging. As individuals age, changes in key metabolic processes such as mitochondrial dysfunction, insulin resistance and altered lipid metabolism disrupt cellular functions and promote systemic inflammation, accelerating aging and age-related diseases. With age, mitochondrial function declines, leading to increased reactive oxygen species (ROS) production and oxidative stress. This damage activates inflammatory pathways and contributes to cellular dysfunction. Insulin resistance, a hallmark of aging, impairs glucose metabolism, elevating proinflammatory cytokines such as IL-6 and TNF, further fueling inflammation. Additionally, visceral fat accumulation during aging leads to the release of proinflammatory adipokines, exacerbating both metabolic dysfunction and inflammation. The release of DAMPs from damaged cells also contributes to the inflammatory environment. DAMPs are recognized by pattern recognition receptors on immune cells, triggering an inflammatory response that exacerbates the chronic low-grade inflammation typical of aging^43,46^. The continuous activation of immune cells by DAMPs accelerates tissue damage and immune dysfunction, further promoting aging and age-related diseases. The interplay between metabolic dysregulation and inflammation accelerates the onset and progression of age-related diseases, including cardiovascular diseases, neurodegeneration, type 2 diabetes and osteoarthritis. DAMPs, released due to cellular stress, further impair tissue repair mechanisms and immune function, compounding the effects of aging and making tissues more susceptible to these diseases^44,45^. This chronic inflammatory environment not only exacerbates the damage but also contributes to the decline in immune system efficiency, increasing vulnerability to infections and reducing the efficacy of vaccines in the elderly.

#### Gut microbiome alterations

Aging induces important changes in the gut microbiome, including reduced microbial diversity and increased prevalence of pathogenic species, disrupting gut homeostasis and amplifying systemic inflammation. This dysbiosis impairs intestinal barrier function, leading to increased permeability or ‘leaky gut’, which allows bacterial translocation and triggers inflammaging. Elevated proinflammatory cytokines from this process contribute to age-related diseases such as cardiovascular and neurodegenerative disorders^59^. Beneficial bacteria, including Akkermansia and Butyricimonas, are diminished in aging individuals, while pathogenic species become dominant, reducing immune resilience and increasing susceptibility to infections^60^. Additionally, a decline in short-chain fatty acid-inducing bacteria exacerbates inflammation and weakens immune responses^61,62^. Dysbiosis activates immune cells through DAMPs released from damaged tissues, perpetuating chronic inflammation and immune dysfunction^63^. Interventions such as prebiotics, probiotics, dietary modifications and fecal microbiota transplantation promise to restore healthy microbiota. Prebiotic fibers, such as galacto-oligosaccharides, promote beneficial bacteria, enhance gut barrier function and reduce inflammation, offering therapeutic potential for mitigating dysbiosis and improving immune function in aging populations^62,64^.

### Interplay between immunosenescence and inflammaging

Immunosenescence and inflammaging are interconnected hallmarks of immune aging that drive systemic dysfunction and age-related diseases. Immunosenescence involves declining immune surveillance and response capacity while inflammaging perpetuates chronic, low-grade inflammation through persistent immune activation. Together, these processes create a feedback loop that exacerbates age-related pathology.

NK cells illustrate this complexity. Systemically, NK cells show reduced cytotoxicity and cytokine production, impairing their ability to clear pathogens and senescent cells. Locally, in SASP-influenced tissues, NK cells paradoxically amplify inflammation, worsening tissue damage^65–70^. Similarly, macrophages display impaired antigen presentation systemically but polarize toward a proinflammatory M1 state in SASP-driven environments^71,72^.

The site and mode of inflammatory stimulation also shape immune outcomes. Systemic sites, such as lymph nodes or blood, exhibit generalized immune activation. At the same time, localized signals in tissues such as the liver or lungs trigger responses that disrupt metabolic homeostasis without robust adaptive immunity^73,74^. These differences underscore the importance of tailoring inflammatory modulation strategies to target specific sites using nanoparticle delivery or site-selective cytokine modulators.

T cells further exemplify the duality of immune aging. Senescent T cells release chronic inflammatory cytokines in tissues, exacerbating damage, whereas T_reg_, which increase with age, suppress adaptive immunity systemically but may mitigate local inflammation^69,71^.

In summary, immunosenescence and inflammaging reinforce each other, destabilizing immune homeostasis in aging. Effective therapeutic strategies must rebalance these processes by enhancing immune functionality and suppressing maladaptive inflammation to mitigate age-related diseases and extend healthspan.

## Immune aging recovery technologies

**Fig. 2:**
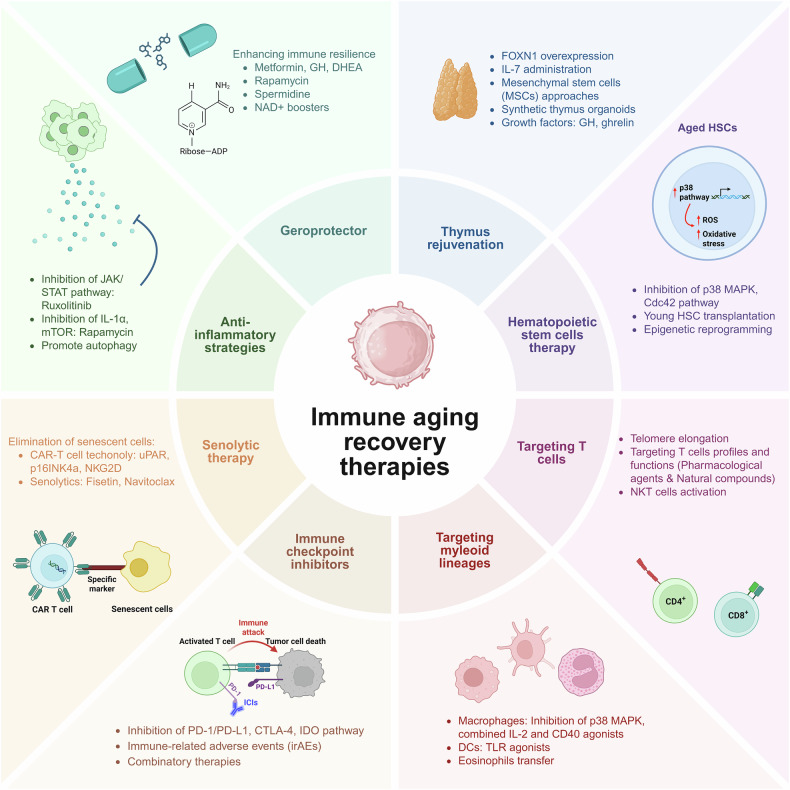
Immune aging recovery therapies. Therapeutic strategies targeting immune aging include thymus rejuvenation, HSC therapy, T cell modulation, myeloid lineage regulation, ICIs, senolytic approaches, anti-inflammatory interventions and geroprotectors. These therapies aim to restore immune resilience, reduce chronic inflammation and enhance systemic health to combat aging-related decline. The figure was created with BioRender.com.

Various therapeutic strategies have been developed to restore immune balance and enhance immune resilience to address the challenges posed by immune aging. These technologies target key mechanisms of immune dysfunction, such as thymic involution, HSC aging and chronic inflammation, while leveraging advances in cellular rejuvenation and immune modulation. Figure 2 summarizes the notable approaches in immune aging recovery, including their mechanisms, therapeutic applications and representative examples.

### Thymus rejuvenation

Recent advances focus on strategies to restore thymic function and improve immune health in aging populations. FOXN1, a key transcription factor for thymic maintenance, has shown potential in reversing thymic involution. Overexpressing FOXN1 restores thymic architecture and enhances T cell production, with studies demonstrating the ability of FOXN1-reprogrammed fibroblasts to generate functional thymic structures^75^. IL-7, a cytokine critical for immune rejuvenation, enhances thymopoiesis and improves T cell output following bone marrow transplantation in aged mice. Whereas IL-7 therapy partially restores thymic function in older mice, further research is needed to explore its human applications^76,77^.

Mesenchymal stem cells offer anti-inflammatory and regenerative properties, homing to thymic injury sites to promote repair and improve function. This approach has shown promise in restoring thymic output and supporting immune health in aged models^78^.

Innovative approaches include synthetic thymus models and bioengineered thymus organoids, which replicate the thymic microenvironment to support T cell maturation and lymphopoiesis. These techniques have potential applications in treating thymic dysfunction and immunodeficiency disorders^78,79^. Additionally, growth factors such as growth hormone and ghrelin stimulate thymic regeneration, enhancing thymopoiesis and immune function in aged models, further highlighting their utility in thymic rejuvenation therapies^80^.

### Cellular rejuvenation therapies of HSCs

Cellular rejuvenation therapies aim to restore the function of aging HSCs, which are essential for maintaining blood and immune health. Targeting the p38 MAPK and Cdc42 pathways, key contributors to HSC aging, has shown promise. Elevated p38 MAPK activity increases ROS levels, impairing HSC function, while pharmacological inhibition reduces ROS, rejuvenates aged HSCs and enhances regenerative capacity. Similarly, inhibiting elevated Cdc42 activity restores HSC polarity and function to a youthful state^81–84^. Bone marrow transplantation is another effective strategy, with studies showing that young donor HSCs extend lifespan and improve systemic health in aged recipients. Additionally, CD22 antibody blockade, targeting a negative regulator of microglial phagocytosis, restores immune cell function and cognitive performance in aged mice, highlighting its potential for immune rejuvenation^85–87^. Epigenetic modifications, including reprogramming DNA methylation and histone patterns, have also demonstrated efficacy in restoring HSC self-renewal and differentiation capacity. These approaches rejuvenate aging HSCs and improve their functional potential^88,89^. These findings underscore the potential of targeting molecular pathways, transplantation and epigenetic reprogramming to combat age-related HSC decline, paving the way for therapies that enhance healthspan and resilience.

### T cell therapy

T cell therapy has emerged as a promising approach to mitigate immune aging and enhance immune resilience in older populations^90^. Telomere elongation is a key focus, with antigen-presenting cells transferring telomeres to CD4^+^ T cells to prevent senescence and enhance immune memory. This process, independent of telomerase, involves telomere-containing vesicles and proteins such as TZAP and Rad51, delaying T cell aging and providing long-term immune protection. Further research is needed to explore its applicability to other immune cells^35,91–94^. Regulating CD4^+^ T cell subsets plays a pivotal role in mitigating immunosenescence. Agents like mTOR inhibitors (for example, RAD001) reduce PD-1^+^CD4^+^ T cells and restore immune functionality, while Dasatinib and Quercetin reverse aging-related T cell dysfunction by modulating differentiation and reducing FoxP3^+^ T_reg_^95,96^. Similarly, checkpoint blockade therapies targeting PD-1/PD-L1 and CTLA-4 increase proliferation in PD-1^+^TCF-1^+^ (Tim-3^−^) stem-like CD8^+^ T cells, improving immune surveillance and control over infections and cancers^97^. NK T cells, which decline with age, can be enhanced through glycolipid-based therapies, improving immune resilience and reducing disease susceptibility^98^. Senolytic immunotoxins such as Anti-HCD2-SAP reduce senescent T cells, while mitochondria-targeted antioxidants such as SkQ1 restore the CD4^+^/CD8^+^ ratio, which is critical for immune balance^99,100^. Metformin also contributes to immune homeostasis by modulating T cell metabolism and reducing proinflammatory Th17 cells^101^. Natural compounds such as icariin and resveratrol show promise in T cell therapy. Icariin improves immune function by modulating T helper cell differentiation and reducing inflammatory cytokines, while resveratrol boosts thymic function and T cell numbers^102,103^. Additionally, CD153-targeted vaccination reduces senescent T cells, improving metabolic balance in aging and obesity-related disorders^104^. These approaches demonstrate the potential of T cell therapies to restore immune function and promote healthier aging. Further research is essential to optimize these strategies for clinical use in aging populations.

### Modulating myeloid lineage and inflammatory cells

Targeting myeloid lineage and inflammatory cells is an effective strategy to counteract immune dysfunction and chronic inflammation associated with aging. Macrophages, which play a central role in tissue repair and inflammation resolution, exhibit functional impairments with age. Elevated p38 MAPK activity in aging macrophages reduces their ability to clear apoptotic cells, contributing to unresolved inflammation. Pharmacological inhibition of p38 MAPK has been shown to restore macrophage function, enhance apoptotic cell clearance (efferocytosis) and promote a proresolving phenotype, which is critical for maintaining tissue integrity in aging populations^105,106^. Additionally, combining IL-2 with CD40 agonists reactivates macrophages, increasing their capacity to clear senescent cells and reduce proinflammatory signals. This dual approach not only boosts macrophage functionality but also rebalances pro- and anti-inflammatory factors, mitigating chronic inflammation and promoting systemic immune rejuvenation^107^. DCs, as key antigen-presenting cells, are essential for initiating and modulating immune responses. However, aging diminishes their ability to activate T cells and respond effectively to pathogens. TLR agonists have emerged as potent activators of conventional DC subsets, such as cDC2, improving their antigen presentation capabilities and modulating inflammation. TLR-based adjuvants have demonstrated efficacy in enhancing vaccine responses and immune resilience in older populations, making them promising tools for restoring DC functionality^108,109^. Eosinophils, traditionally recognized for their role in allergic reactions, are increasingly acknowledged for their contribution to tissue homeostasis and immune regulation. Studies have shown that transferring eosinophils from young to aged animals can restore tissue balance, enhance immune surveillance and reduce systemic inflammation. These findings highlight the therapeutic potential of eosinophil-based interventions for improving immune function in aging populations^110,111^.

### Immune checkpoints and rejuvenation strategies

Immune checkpoint inhibitors (ICIs) are revolutionizing cancer immunotherapy by restoring the immune system’s ability to combat tumors, even in aging populations. ICIs work by blocking inhibitory signals such as PD-1/PD-L1 and CTLA-4, which are often upregulated in older adults and contribute to immune suppression^112^.

Aging introduces additional challenges to ICI efficacy, including reduced tumor-infiltrating lymphocytes and increased T cell exhaustion mediated by inhibitory receptors such as PD-1, Tim-3 and LAG-3. Despite these barriers, studies have shown that ICIs can achieve comparable efficacy in older and younger patients. For example, a meta-analysis of patients with non-small cell lung cancer revealed similar therapeutic outcomes across age groups, underscoring the feasibility of ICIs in aging populations^113,114^.

However, the immunosuppressive tumor microenvironment, enriched with SASP factors such as IL-6 and TGF-β, remains a considerable obstacle. While IL-6 is a prominent SASP cytokine that can reinforce senescence via autocrine and intracrine signaling, thereby promoting tumor growth and immune evasion in both senescent and neighboring cells, TGF-β contributes by inducing and sustaining the senescent phenotype as well as further suppressing antitumor immune responses^44,115,116^. Anti-SASP therapies, targeting cytokines such as IL-6 and TGF-β, have shown promise in reducing tumor microenvironment immunosuppression and enhancing ICI efficacy. Additionally, combining ICIs with CAR-T cell therapy or oncolytic viruses further amplifies cytotoxic T cell responses in SASP-enriched environments^117,118^.

To address the decline in T cell functionality due to immunosenescence, strategies such as telomere elongation and checkpoint pathway optimization are being explored. These approaches aim to rejuvenate exhausted T cells and expand the naive T cell pool, directly enhancing antitumor immunity. Improving tumor-infiltrating lymphocyte recruitment through chemokine-based therapies or engineered interventions further complements these strategies^119^.

Although ICIs are generally well tolerated, older patients may experience immune-related adverse events such as myocarditis, encephalitis or myasthenia gravis. Effective management strategies, including the use of predictive biomarkers and personalized treatment plans, are crucial to optimizing safety and efficacy in this demographic. Tailoring ICI therapies based on individual patient profiles ensures that age-related risks are minimized while therapeutic potential is maximized^120^.

### Senolytic and anti-inflammatory strategies

Senolytic therapies, which selectively eliminate senescent cells, and anti-inflammatory strategies that suppress the harmful effects of the SASP are increasingly recognized for their ability to extend healthspan and lifespan. Chimeric antigen receptor (CAR) T cell technologies, designed to target senescent cell markers such as urokinase-type plasminogen activator receptor (uPAR) and p16INK4a, have shown efficacy in preclinical models. These CAR-T cells selectively clear senescent cells, reducing systemic inflammation and restoring tissue homeostasis^121,122^. In addition to CAR-T therapies, small-molecule senolytics such as fisetin, a natural flavonoid, and navitoclax (ABT263), a BCL-2 family inhibitor, have demonstrated effectiveness in removing senescent cells and mitigating SASP-driven inflammation. These interventions rejuvenate HSCs, enhance immune function and support tissue repair^123,124^. Especially, small clinical trials have tested intermittent high-dose regimen of fisetin in osteoarthritis, frailty and cardiovascular disease populations, showing enhanced physical function and decreased SASP factors (IL-6 and CRP), though effects vary by disease subtype and baseline senescence burden^125^. Other clinal studies further highlight senolytics as a promising strategy to counteract immune aging and improve outcomes in age-related infections such as COVID-19. Randomized clinical test of Quercetin in patients with mild COVID-19 demonstrated the reductions in hospitalization rates, oxygen needs, ICU admission and mortality, with no big side effects^126^. In a pilot study of dasatinib plus quercetin in idiopathic pulmonary fibrosis, senolytic treatment was generally well tolerated and improved physical capability, supporting the potential of senolytics to mitigate fibrosis, a complication relevant to severe COVID-19 (ref. ^126^). Anti-inflammatory approaches, including JAK/STAT inhibitors such as ruxolitinib, directly modulate SASP production, alleviating chronic inflammation and improving tissue integrity. Similarly, mTOR inhibitors such as rapamycin reduce proinflammatory activity and promote tissue regeneration by regulating SASP components such as IL-1α. Emerging evidence also highlights the role of autophagy induction in suppressing SASP-mediated inflammation and restoring cellular homeostasis in aging models^47,127,128^. By combining targeted senescent cell clearance with SASP modulation, senolytic and anti-inflammatory strategies address the dual challenges of immune dysfunction and chronic inflammation, offering great potential for improving healthspan and mitigating aging-related diseases.

### Enhancing immune resilience through geroprotective interventions

Geroprotective interventions, long recognized for extending lifespan, are increasingly being validated for their potential to improve immune function and mitigate immunosenescence. Emerging evidence demonstrates that established geroprotective strategies enhance the lifespan and play a critical role in restoring immune balance, promoting both lifespan and healthspan. The combination of growth hormone (GH), dehydroepiandrosterone (DHEA) and metformin has shown promising results in reversing epigenetic aging and improving immune function. Fahy et al.^129^ demonstrated that this combination induces thymic regeneration, restoring T cell production and diversity. Enhanced thymic function reduces systemic inflammation and rejuvenates immune surveillance, offering a dual benefit for both aging and immune recovery. This intervention provides a foundation for addressing immunosenescence in humans. Metformin, a widely used antidiabetic drug, has been found to modulate immune homeostasis by reducing proinflammatory cytokines and improving macrophage and T cell functionality. Aspinall and Lang^130^ highlighted metformin’s role in mitigating systemic inflammation and enhancing the immune response in aged populations. Similarly, rapamycin, an mTOR inhibitor, modulates chronic inflammation and restores the functional capacity of aging immune cells^95^. Low-dose rapamycin improves vaccine responses in older adults, demonstrating its efficacy in counteracting immune decline.

Among geroprotective strategies, mTOR inhibition by rapamycin has been validated not only for its systemic anti-aging effects but also for its direct impact on immune aging. In a randomized, placebo-controlled phase 2a clinical trial (NCT03373903), a combination of catalytic (BEZ235) and allosteric (RAD001) mTOR inhibitors was administered to 264 elderly individuals^131^. This regimen markedly reduced the incidence of respiratory tract infections during the winter season, a period of heightened immune vulnerability in the elderly.

Mechanistically, the treatment upregulated antiviral gene expression, indicating enhanced innate immune preparedness. In a phase 3 trial (NCT04668352), the TORC1 inhibitor RTB101 was administered to 1024 adults aged 65 years and older to evaluate its efficacy in preventing clinically symptomatic respiratory illnesses. Wheras RTB101 did not significantly reduce the incidence of symptomatic respiratory infections compared with placebo, it did induce a significant upregulation of interferon-stimulated antiviral gene expression, indicating enhanced innate immune priming in elderly participants^132^. These molecular effects suggest that TORC1 inhibition may modulate age-related immune dysfunction even in the absence of overt clinical benefits, supporting its potential as an immunomodulatory geroprotective strategy in aging populations.

Spermidine, a natural polyamine, activates autophagy, a critical process for maintaining cellular homeostasis. Bravo-San Pedro et al.^133^ revealed that spermidine enhances immune cell function by clearing damaged cellular components, thereby supporting robust immune responses in aging populations. NAD^+^ boosters, which restore mitochondrial function, have also demonstrated the potential to improve immune cell energetics^134^. Restoring mitochondrial efficiency enables immune cells to better respond to stress and maintain surveillance, further reinforcing immune resilience^135^. The importance of these interventions became particularly evident during the COVID-19 pandemic. Geroprotective strategies such as rapamycin, metformin and NAD^+^ boosters reduced the severity and lethality of infections and provided insights into addressing the broader challenges of aging immune systems^7^. While current agents such as rapamycin, metformin and NAD⁺ boosters have demonstrated the ability to partially restore immune function and enhance antiviral responses in aged populations, their clinical benefits remain modest and context-dependent. These limitations highlight the need for next-generation immunomodulatory strategies that more precisely target the core mechanisms of immune aging. The development of such targeted interventions will be essential for achieving robust and durable reversal of immunosenescence, thereby supporting not only immune competence but also systemic health and longevity.

## Promoting lifespan extension through balanced inflammation regulation

In the previous sections, we discussed how aging of the immune system—driven by thymic involution, HSC dysfunction and the accumulation of senescent immune cells—contributes to immunosenescence and inflammaging. These processes lead to impaired immune surveillance, chronic low-grade inflammation and increased vulnerability to infections, cancer and tissue degeneration. We also introduced several emerging strategies to reverse or mitigate immune aging at the cellular and tissue levels, such as thymic regeneration, mitochondrial rejuvenation and clearance of senescent immune cells.

**Fig. 3:**
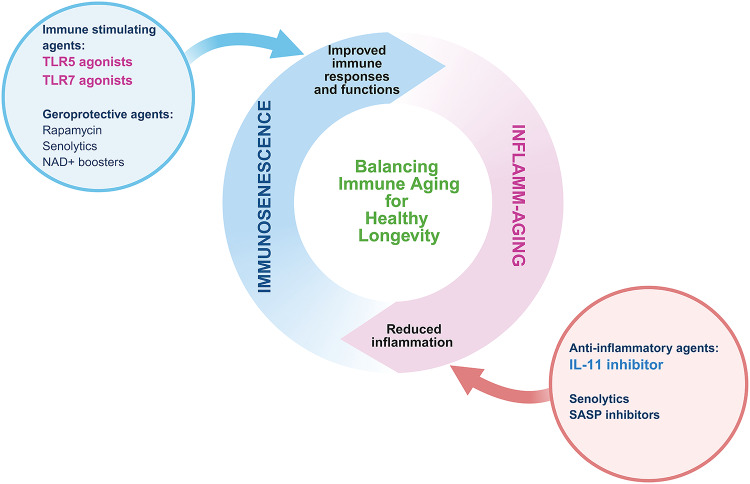
The interplay between inflammaging and immunosenescence in the context of aging and longevity. Inflammaging represents the chronic low-grade inflammation observed during aging, contributing to frailty and age-related diseases, which can be mitigated through anti-inflammatory agents such as IL-11. Conversely, immunosenescence refers to the decline in immune function, characterized by reduced pathogen defense and impaired cancer surveillance, which can be addressed using immune-stimulating agents such as TLR5 or TLR7 agonists and geroprotective agents such as rapamycin, metformin and NAD⁺. Balancing these processes can promote healthy longevity by reducing inflammation-induced frailty while enhancing immune resilience against infections and cancers. The central strategy emphasizes the dual modulation of inflammatory and immune pathways to optimize healthspan and lifespan outcomes. The figure was created with BioRender.com.

Here, we extend the discussion beyond cellular restoration to ask a broader question: Can immune modulation itself serve as a lever to control organismal aging? Rather than merely targeting downstream damage, we explore how regulating immune balance—by suppressing excessive inflammation or enhancing immune function—can influence aging at a systemic level. Immune cells orchestrate inflammation, regeneration and tissue homeostasis across organs; thus, fine-tuning immune responses may enable control over age-associated functional decline and lifespan itself. Interestingly, recent studies have shown that both enhancing immune activity and suppressing chronic inflammation can directly modulate systemic aging. Such interventions have been associated with increased resistance to age-related diseases, recovery of physiological functions and even lifespan extension in preclinical models. These findings suggest that technologies targeting immunosenescence and inflammaging are not merely supportive but may act as true geroprotectors—agents capable of delaying or reversing the biological aging process. Here, we highlight recent strategies that aim to recalibrate immune responses to achieve healthy longevity, including the use of anti-inflammatory compounds, immunostimulatory adjuvants and cytokine modulators such as IL-7, IL-11 inhibitors and TLR agonists. We discuss how these immune-targeted interventions integrate upstream with the mechanisms of immunosenescence and relevant recovery technologies, offering a coherent framework for aging control through immune regulation. Figure 3 illustrates the intricate balance between inflammaging and immunosenescence in the context of aging and longevity. Inflammaging contributes to frailty and age-related diseases, which can be mitigated through anti-inflammatory interventions. Conversely, immunosenescence leads to immune decline, which can be addressed by strategies to improve immune function. The figure emphasizes that balancing these processes is key to promoting healthspan and lifespan by reducing inflammation-induced damage while enhancing immune resilience against infections and cancers. This dual-modulation approach provides a foundation for developing effective therapies to combat aging-related functional decline.

### Suppressing inflammation for lifespan extension

One promising avenue is the suppression of specific proinflammatory cytokines, such as IL-11, to mitigate chronic inflammation and support tissue regeneration. IL-11 has been identified as a key mediator in promoting fibrosis and systemic inflammation, particularly in cardiovascular and aging contexts^136,137^. Therapeutic strategies targeting IL-11 signaling have demonstrated crucial potential in reversing fibrosis and alleviating systemic inflammation while enhancing tissue-specific repair. For instance, IL-11 inhibition in cardiovascular fibrosis models reduced inflammatory markers and promoted tissue homeostasis, presenting a novel approach for addressing age-related inflammatory diseases^138,139^.

In aging, IL-11 overexpression contributes to pathological conditions such as cardiovascular fibrosis and systemic inflammation, exacerbating the effects of inflammaging. Notably, the pharmacological and genetic blockade of IL-11 has improved lifespan and healthspan in preclinical models, underscoring its therapeutic relevance. These findings highlight that targeted cytokine modulation, rather than broad suppression of all inflammatory pathways, is critical for promoting healthy aging and minimizing the adverse effects of systemic immune suppression. Recent studies have also shown that IL-11 inhibition can potentially extend healthspan and lifespan. Specifically, blocking IL-11 signaling in mammalian models has resulted in marked improvements in both tissue repair and systemic health, ultimately contributing to longevity and enhanced vitality^8^.

### Stimulating innate immunity for lifespan extension

Equally critical is the activation of mucosal immunity to maintain immune surveillance and systemic immune resilience. TLR5 has gained recognition as a key target for aging-related interventions, addressing both immunosenescence and inflammaging. Unlike other TLRs, TLR5 maintains its expression and functionality during aging, providing a distinct opportunity to enhance immune responses, promote tissue regeneration and extend lifespan. Recent studies have demonstrated the profound impact of TLR5 activation on longevity. Using flagellin, a natural agonist, to repeatedly stimulate TLR5 through nasal mucosal pathways, researchers observed prominent improvements in immune function and reductions in aging-associated decline. This intervention modulated inflammatory responses, enhanced immune surveillance and ultimately extended lifespan with a broad range of beneficial health effects in aging models^9^.

TLR5 also plays a pivotal role in improving immune activation, particularly in overcoming the reduced vaccine efficacy associated with aging. Preclinical studies have shown that flagellin enhances antibody responses to seasonal influenza vaccinations, acting as a mucosal adjuvant to boost immune function. This approach restores vaccine efficacy in older populations, addressing a key challenge of immunosenescence^140–145^. Furthermore, TLR5 activation strengthens mucosal immunity, an essential barrier against infections in aging individuals.

Beyond immune activation, TLR5 signaling has contributed to tissue regeneration and metabolic health. TLR5-deficient models exhibit severe metabolic disturbances due to microbiota dysregulation, underscoring its importance in maintaining metabolic and immune balance^146^. Studies have also demonstrated that TLR5 activation induces IL-22, a cytokine essential for epithelial barrier integrity and tissue repair^147,148^. This pathway has been shown to enhance tissue regeneration, particularly in the liver, and improve overall metabolic function, making TLR5 a promising therapeutic target for addressing age-related dysfunction and metabolic disorders^149,150^.

In addition to TLR5, TLR7 is a key innate immune sensor located in endosomes that recognizes single-stranded RNA from viruses such as SARS-CoV-2 and activates downstream proinflammatory and antiviral responses through type I interferon and cytokine production^151^. However, TLR7 activity declines with age, impairing immune responses and contributing to immunosenescence^152^. Notably, Triana-Martinez et al.^153^ have reported the activation of TLR7 with the BNT162b2 mRNA COVID-19 vaccine rapidly induces p16^High^ immune cells (including T_reg_ and macrophages). These cells play a central role in establishing disease tolerance and protecting tissues during infection by lowering adenosine levels, maintaining tonic STING signaling and balancing immune responses by resolving inflammation (via IL-10/Arg1) without triggering harmful cytokine storms. Controlled TLR7 stimulation restores beneficial p16^High^ immune populations that typically decline with age, thereby counteracting immunosenescence. This effect is also seen in MDA5-deficient mice, which maintain protective p16^High^/STING activity and exhibit improved healthspan^153^. These findings suggest that TLR7-targeted strategies-like mRNA vaccines-can enhance early immune protection and promote tissue resilience in older adults, offering a promising approach to mitigate age-related immune decline^151,153,154^.

## Conclusion and perspective

Aging profoundly affects the immune system, leading to two interrelated phenomena: immunosenescence and inflammaging. Immunosenescence is characterized by the immune system’s functional decline, reduced immune surveillance, diminished T cell diversity and a weakened response to new infections and vaccinations. Inflammaging, on the other hand, refers to chronic, low-grade inflammation driven by factors such as senescent cells, SASP, DAMPs and alterations in the gut microbiome. Together, these processes accelerate tissue degeneration, systemic dysfunction and the development of age-related diseases while further impairing immune function.

Emerging therapeutic strategies targeting immunosenescence and inflammaging offer hope for restoring immune balance, reducing inflammation and extending healthspan. Interventions such as thymus rejuvenation, HSC modulation and senolytic therapies can potentially combat immune decline. Additionally, technologies targeting IL-11 inhibition and TLRs (TLR5 or TLR7) activation have effectively reduced chronic inflammation and enhanced immune resilience. Specifically, IL-11 inhibition mitigates systemic inflammation and supports tissue regeneration, while TLR5 or TLR7 activation strengthens immune function and promotes regenerative capacity, collectively contributing to lifespan extension.

However, understanding the complexity of immunosenescence and inflammaging is critical to developing effective therapeutic interventions. While chronic inflammation is often viewed as detrimental, inflammation plays a vital role in immune defense, tissue repair and vaccine efficacy. The challenge lies in maintaining a balance—promoting inflammation’s protective effects while mitigating its chronic, maladaptive impacts during aging.

Future research must focus on refining these therapeutic strategies and transitioning them from preclinical to clinical applications. Personalized medicine, guided by genetic and immune profiling, offers an opportunity to tailor interventions for maximum benefit. Promising approaches include targeting senescent cells, modulating the gut microbiome and leveraging immune checkpoint modulation and epigenetic reprogramming.

Ultimately, by addressing both immune decline and chronic inflammation, these strategies can potentially transform how aging and age-related diseases are managed. Success in these endeavors could extend lifespan and meaningfully improve healthspan, ensuring healthier aging for future generations.
